# Exploiting Superpixels for Multi-Focus Image Fusion

**DOI:** 10.3390/e23020247

**Published:** 2021-02-21

**Authors:** Areeba Ilyas, Muhammad Shahid Farid, Muhammad Hassan Khan, Marcin Grzegorzek

**Affiliations:** 1 Punjab University College of Information Technology, University of the Punjab, Lahore 54000, Pakistan; areeba.ilyas@pucit.edu.pk (A.I.); hassankhan@pucit.edu.pk (M.H.K.); 2 Institute of Medical Informatics, University of Lübeck, Ratzeburger Allee 160, 23538 Lübeck, Germany; grzegorzek@imi.uni-luebeck.de

**Keywords:** multi-focus image fusion, image enhancement, information fusion color distance models for fusion

## Abstract

Multi-focus image fusion is the process of combining focused regions of two or more images to obtain a single all-in-focus image. It is an important research area because a fused image is of high quality and contains more details than the source images. This makes it useful for numerous applications in image enhancement, remote sensing, object recognition, medical imaging, etc. This paper presents a novel multi-focus image fusion algorithm that proposes to group the local connected pixels with similar colors and patterns, usually referred to as superpixels, and use them to separate the focused and de-focused regions of an image. We note that these superpixels are more expressive than individual pixels, and they carry more distinctive statistical properties when compared with other superpixels. The statistical properties of superpixels are analyzed to categorize the pixels as focused or de-focused and to estimate a focus map. A spatial consistency constraint is ensured on the initial focus map to obtain a refined map, which is used in the fusion rule to obtain a single all-in-focus image. Qualitative and quantitative evaluations are performed to assess the performance of the proposed method on a benchmark multi-focus image fusion dataset. The results show that our method produces better quality fused images than existing image fusion techniques.

## 1. Introduction

Due to limited depth-of-field (DOF), it is not easy for cameras to capture an image that has all focused objects. Usually, in a camera captured image, the objects that lie in the range of the depth-of-field are sharp and the remaining objects tend to be blurred [1]. An additional cause of image blurring includes atmospheric scatter and spatial and temporal sensor integration [2]. Images that are partially focused are not enough for sound understanding and obtaining accurate results in different applications of computer vision such as object recognition and extraction [3], remote sensing and surveillance [4], image enhancement [5], medical imaging [6], etc. To resolve this issue, multi-focus image fusion algorithms are proposed in which a fused image of an extended depth-of-field is constructed by integrating the additional information of multiple images of the same scene.

In recent years, numerous multi-focus image fusion methods have been proposed. Some of them directly operate on image pixels and regions to obtain an all-in-focus image while others exploit the transform domains to achieve this task. The former methods are known as spatial domain based fusion methods, and the latter are categorized as transform domain based methods [7,8]. In transform domain based fusion, a fused image is regenerated from fused transform coefficients of the source images. The transform domain based fusion methods perform well for separating the discontinuities at edges, but do not give the representation for line and curve discontinuities [9]. They are computationally expensive techniques and may produce undesirable artifacts in fused image [10].

In spatial domain based fusion, the rules of fusion are applied to pixels or a region of input images directly without conversion to other representations to generate the fused image. Most spatial domain fusion algorithms operate at the pixel level, e.g., [11,12,13,14], and produce a fused image by weighting the corresponding pixels of source images. Some spatial domain based fusion techniques work at the region level, e.g., [15,16,17]. These methods compute focus measures for each region and construct a fused image by selecting a region with more clarity. The spatial domain based fusion algorithms provide high spatial resolution. However, the results might suffer from unwanted artifacts, e.g., the ghosting effect, which mainly occurs due to incorrect categorization of the pixels or regions as focused or de-focused. To overcome these problems, some techniques in the spatial domain use real values instead of binary values as focus measures and use the weighted averaging of pixel values to obtain the fused image [18,19].

**Figure 1:**
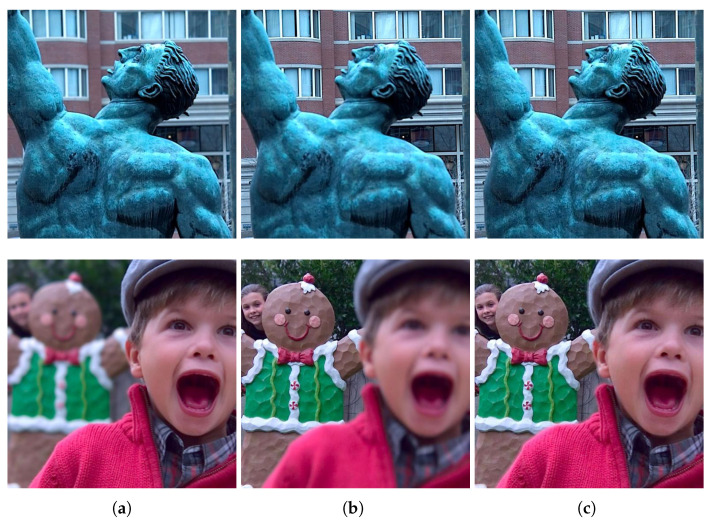
Results of the proposed algorithm on sample multi-focus images from the test dataset. (**a**) Source images with foreground focused, (**b**) source images with background focused, and (**c**) fused images using the proposed method.

In this paper, we propose a novel spatial domain based multi-focus image fusion algorithm. The proposed method is built on the idea of grouping the neighboring pixels with similar colors and patterns into larger pixels (also known as superpixels) to obtain an accurate decision map. We observe that such a grouping helps reduce the false categorization of pixels as focused and de-focused. The statistical properties of the computed superpixels are analyzed to distinguish the focused and de-focused pixels, generating the initial focus map, which is further processed to obtain a more accurate final decision map. Different tests performed to evaluate the performance of the proposed method reveal its effectiveness. A few of the sample fusion results achieved by the proposed method are shown in Figure 1.

The rest of this paper is organized as follows: A brief review of the literature on multi-focus image fusion is presented in Section 2. The proposed MIFalgorithm is presented in Section 3. The experimental evaluation through different qualitative and quantitative tools is carried out in Section 4. The performance of the proposed algorithm with different color distance models and the impact of other parameters is analyzed in Section 5. The conclusions are drawn in Section 6.

## 2. Background and Literature Review

The main step in any multi-focus image fusion algorithm is the detection of the focused region in the source multi-focus images to obtain a so-called decision map, also called the focus map. The source images are then fused to obtain an all-in-focus image. Based on the representation and working of the focus map estimation and fusion process, the existing MIF algorithms can be categorized into four groups: multi-scale transform based methods, feature space transform based methods, spatial domain based methods, and neural networks based methods [8,20].

The multi-scale transform based methods generally split the input images into a multi-scale domain and then fuse the transformed coefficients using some fusion rule. The all-in-focus image is generated from these fused coefficients [21]. Multi-scale transform methods have a wide of range of related techniques sequentially implemented in image fusion like pyramid decomposition [22], wavelet transform [23,24], curvelet transform (CVT) [25], and contourlet transform [26,27]. In the MIF algorithm presented in [28], the source images are split into approximation and detail coefficients at different levels, and the coefficients are fused by applying several fusion rules. Various level fused coefficients are merged to obtain the desired all-in-focus image. Sheng et al. [29] proposed a support value transform based method that uses a support vector machine to achieve the fused image. The discrete cosine transform has also been exploited for image fusion, e.g., [30,31]

The feature space transform based fusion has emerged as another popular mean for multi-focus image fusion [32]. These fusion methods estimate the focus map through a single scale feature space of source images. References [1,13,33,34] are a few examples of such MIF methods. The sparse representation based fusion method in [33] divides the input images into patches known as sparse coefficients using a dictionary. The coefficients are then combined, and the fused image is generated. The fusion method in [1] uses the feature space from a robust principal component analysis technique for decomposition, and local sparse features are used to compute the all-in-focus image. The higher order singular-value decomposition based method [34] picks informative patches from input images by estimating the activity level of each patch. This information helps to obtain the fused image. Another higher order singular-value decomposition based method was presented in [35]. Multi-focus image fusion with the dense scale-invariant feature transform method (DSIFT) [13] uses dense descriptors to calculate the activity level in the patches of source images to form an initial decision map. After refining and feature matching of the initial map, it is used to form the fused image.

In spatial domain based fusion methods, fused images are computed by processing the pixels of the input multi-focus images. Some methods operate at the pixel level, e.g., [11,14,36]; some are block based, e.g., [37,38,39]; and others are region based methods, such as [40,41,42]. Usually, pixel-by-pixel averaging of source images does not achieve plausible results as it may introduce different artifacts in the fuse image [13,43]. Therefore, the region based and block based spatial domain fusion methods have been introduced. In these methods, the input images are divided into blocks or segmented regions, and the sharpness of each block is calculated to form the focus map [44].

Image segmentation is a difficult task, so it is hard to maintain the quality of fused images obtained by region based methods. However, as an image block may contain both focused and defocused areas, the fused image computed by the block based method may exhibit anisotropic blur and misregistration. To resolve this problem, multi-scale image fusion methods have been introduced. The method in [45] is based on a multi-scale structure based focus measure. In this method, a binary-scale scheme is used, which calculates the weighted gradient of a focused region by applying small-scale focus measures. It proves helpful in reducing the anisotropic blur and misregistration with large-scale focus measures.

In recent years, many pixel based spatial domain methods have been proposed. These methods include image matting based [18] and guided filtering based [46] methods, which compute the fused image by extracting enough information from the source images and achieve high efficiency by preserving spatial consistency. The MIF method proposed in [18] focuses on obtaining all-in-one focus images from detected focused regions in multi-focus images by using morphological filtering and the image matting technique. In the guided filtering based method [46], two-scale image decomposition is done to produce focused images from the base layer and detail layer.

Some pixel based methods, such as [47,48,49], solve the maximum optimization problem by estimating a weight map. The algorithm in [47] has the benefit of a multi-spectral algorithm along with spatial domain methods. The combined activity estimation of high and low frequencies of source images is calculated, and a new smoothness term is introduced for the alignment of the solution to the boundaries of the defocused and focused pixel. In [48], multi-focus image fusion was proposed using edge and multi-matting models. A multi-focus image fusion algorithm based on random walks [49] analyzes the feature area locally and identifies nodes globally to compute connected graphs of random walks.

Multi-focus image fusion has also been achieved through deep neural networks in several ways; the majority of these techniques rely on the detection of the focused region [50]. In the fusion method presented in [50], features are extracted through a u-shaped network to obtain high- and low-level frequency texture information. It directly maps multi-focus images to fused images instead of detecting focused regions. The pulse coupled neural network (PCNN) is also explored for image fusion, e.g., [16]. In these methods, the source multi-focus images are decomposed into fixed-size blocks, and the image Laplacian of each block is computed to obtain the feature maps. These feature maps are used as the input of the PCNN as in [16].

Recently, deep learning has also been explored for efficient image fusion [51]. A number multi-focus image fusion techniques based on deep learning (DL) have been presented using convolutional neural networks (CNNs), such as in [52,53,54,55,56]. The method in [52] proposes a pixel-wise convolution neural network for image fusion, and the deep convolutional neural network (CNN) based method [54] uses the Siamese network for feature extraction and activity level measurement in source images to obtain a focus map. The CNN based MIF method presented in [53] also uses Siamese multi-scale feature extraction. In [55], a multi-level dense network was presented for fusing multi-focus images. It extracts shallow and dense features from images from a mixture of many distributions. The convolutional sparse representation (CSR) is used for multi-focus image fusion, e.g., [57,58,59]. The algorithm in [59] uses Taylor expansion and convolutional sparse representation for fusing multi-focus images. Morphological component analysis and convolutional sparse representation were used in [58] to obtain texture features using pre-learned dictionaries. The obtained sparse coefficients of the source images were merged, and a fused image was obtained. Generative adversarial networks (GANs) have also been exploited for increasing the depth of field, e.g., [60,61,62].

## 3. Proposed Method

**Figure 2:**
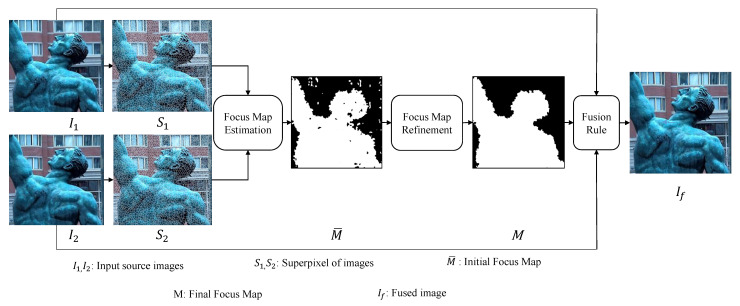
Schematic diagram of the proposed multi-focus image fusion algorithm.

We propose a spatial domain based multi-focus image fusion algorithm that builds on the idea that pixels in a small locality in an image are highly autocorrelated—having similar colors and textures—and thus can be merged into larger patches known as superpixels to improve the speed and quality of different tasks. These superpixels are more expressive than single pixels as they carry more distinctive statistical properties when compared with other superpixels. Moreover, we observe that they have a greater tendency to be distinguished as focused and out-of-focused compared to single pixels. We exploit this important characteristic to design a novel and efficient image fusion algorithm. The proposed method works in three steps. First, superpixels in the source multi-focus images are computed. Second, using the statistical properties of superpixels, they are categorized as focused and de-focused, from which initial focus maps are constructed. The initial focus map is refined using a spatial consistency constraint. Third, the refined focus map is used to obtain the fused image from source multi-focus images with the help of a fusion rule. A schematic diagram of the proposed method is shown in Figure 2. These steps are described in detail in the following sections.

### 3.1. Superpixels Computation

Several algorithms have been proposed to obtain superpixels from an image, e.g., [63,64,65]. Like most existing superpixel algorithms, the proposed technique also uses pixel color and its spatial position in deciding their merging. Our method extends the simple linear iterative clustering (SLIC) algorithm [65], which uses *k*-means clustering to generate superpixels, to obtain superpixels better suited for image fusion. In particular, similar to [65], we divide the image pixels into *k* number of clusters based on their color and location. Superpixels are computed based on the mean color values of pixels. For an image with *N* pixels, the size of each superpixel is approximately N/k pixels. Regular spaced grid pixels *S* are computed where $S=\sqrt{N/K}$. Every initial cluster center is then sampled based on *S* to produce equally sized superpixels. After the comparison, these centers are then moved forward to the lowest gradient position. Then, each pixel of the image is associated with the nearest cluster center through a distance measure $D_{s}$. $D_{s}$ is computed considering the pixel spatial distance $d_{s}$ and the color difference $d_{c}$.

The spatial distance of the pixel located at $\left(x_{i},y_{i}\right)$ and a cluster center $\left(x_{k},y_{k}\right)$ is computed using the Euclidean distance metric.

(1) $$d_{s}=\sqrt{{\left(x_{k}-x_{i}\right)}^{2}+{\left(y_{k}-y_{i}\right)}^{2}}$$

For color distance measure, unlike the method in [65], we use the CIEDE2000color difference model, which produces better superpixels for image fusion. We performed numerous experiments and tested different color distance models to evaluate their effectiveness for pixel focus detection, which favored the CIEDE2000 metric. It is a complicated, but accurate color distance measure [66]. In this model, the color difference between two pixels with color in the $L^{*}a^{*}b^{*}$ model ($L_{1}^{*}a_{1}^{*}b_{1}^{*}$) and ($L_{2}^{*}a_{2}^{*}b_{2}^{*}$) is computed as:

(2) $$d_{c}=\sqrt{{\left(\frac{\Delta L^{{}^{\prime }}}{k_{L}S_{L}}\right)}^{2}+{\left(\frac{\Delta C^{{}^{\prime }}}{k_{C}S_{C}}\right)}^{2}+{\left(\frac{\Delta H^{{}^{\prime }}}{k_{H}S_{H}}\right)}^{2}+R_{T}\frac{\Delta C^{{}^{\prime }}}{k_{C}S_{C}}\frac{\Delta H^{{}^{\prime }}}{k_{H}S_{H}}}$$

where $\Delta L^{\prime }$ is the difference in luminance components and $\Delta C^{\prime }$ and $\Delta H^{\prime }$ are computed from the chroma components $a^{*},b^{*}$.

$$\begin{array}{c} \Delta L^{\prime }=L_{2}^{*}-L_{1}^{*}\\ \Delta C^{\prime }=C_{2}^{{}^{\prime }}-C_{1}^{{}^{\prime }} \end{array}$$

with $C_{1}^{{}^{\prime }}=\sqrt{{\left(a_{1}^{{}^{\prime }}\right)}^{2}+{\left(b_{1}^{*}\right)}^{2}}$, $C_{2}^{{}^{\prime }}=\sqrt{{\left(a_{2}^{{}^{\prime }}\right)}^{2}+{\left(b_{2}^{*}\right)}^{2}}$ and $\Delta H^{{}^{\prime }}=\sqrt{C_{1}^{{}^{\prime }}C_{2}^{{}^{\prime }}}\sin\left(\Delta h{}^{\prime }/2\right)$.

The weighting factor $K_{L}$, $K_{C}$, and $K_{H}$ in (2) are usually set to unity. $S_{L}$, $S_{C}$, and $S_{H}$ are the compensation of lightness, chroma, and hue. To keep the discussion simple, the complete details of the metric are not presented here, and they can be found in [66].

Finally, the two distance measures are combined as in [65] to obtain $D_{s}$. It is the sum of the spatial distances (1) and color distances (2), which are normalized by gird interval *S*. The factor *m* in (3) is used to control the compactness of the superpixels.

(3) $$D_{s}=d_{c}+\frac{m}{S}d_{s}$$

**Figure 3:**
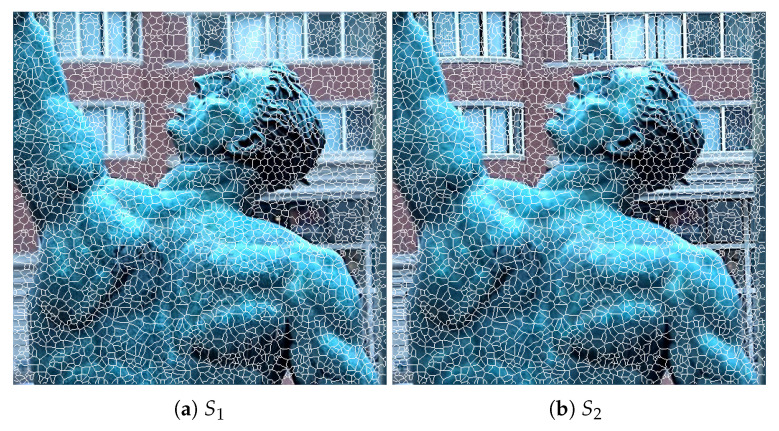
Superpixel computation using the proposed strategy. (**a**) Superpixels of $I_{1}$ (foreground focused). (**b**) Superpixels of $I_{2}$ (background focused).

Let $I_{1}$ and $I_{2}$ be two source multi-focus images. In the rest of the text, they are simply referred to as source images. Using the proposed algorithm, the superpixels in $I_{1}$ are computed, which are mapped to $I_{2}$ to divide $I_{2}$ into the corresponding set of superpixels. That is, the superpixels in $I_{2}$ are not computed; instead, the superpixel labels computed in $I_{1}$ are assumed for $I_{2}$ as well, and the values of their centers are updated with respect to $I_{2}$. This helps to establish the correspondence between the superpixels in $I_{1}$ and $I_{2}$. We recall that both images are complementary and represent the same scene, but with different focused regions. Therefore, the superpixel structure of the two images is kept the same so that the correspondence between them can be established. Figure 3 shows the superpixels computed using the proposed strategy on a sample multi-focus image pair (shown in Figure 1).

### 3.2. Focus Map Estimation

A superpixel carries various statistical properties, e.g., mean, standard deviation, variance, and kurtosis, which can be used to draw different characteristics of the superpixel. The standard deviation is considered to be an important and widely used index to measure information diversity and variability in an image [67,68]. It has been shown in different studies, e.g., [69], that the variance (or standard deviation) of the de-focused regions is generally less than the sharp regions. In the proposed method, we exploit this property to designate the superpixels as focused and de-focused. We calculate the standard deviation of each superpixel in the source images and use it to estimate the focus map. We found that the standard deviation of *L* channel serves as a better discriminant than the standard deviation computed over all three channels, i.e., *l*, *a*, and *b*. Therefore, for each superpixel, *s*, the standard deviation σ of the *L* values of its constituent pixels is computed.

(4) $$\sigma_{1,i}=\sqrt{\frac{1}{\left|s_{1,i}\right|}\sum_{\left(x,y\right)\in s_{1,i}}{\left(I_{1}\left(x,y\right)-\mu_{1,i}\right)}^{2}}$$

where $s_{1,i}$ is the *i*-th superpixel in image $I_{1}$, $\left|s_{1,i}\right|$ is the number of pixels in $s_{1,i}$, and $\mu_{1,i}$ is the mean of the pixels in superpixel $s_{1,i}$.

$$\mu_{1,i}=\frac{1}{\left|s_{i}\right|}\sum_{\left(x,y\right)\in s_{i}}I_{1}\left(x,y\right)$$

The standard deviation of each superpixel in $I_{2}$, $\sigma_{2,i}$, is computed analogously. The standard deviations of the corresponding superpixels in $I_{1}$ and $I_{2}$ are compared, and the focus map $\bar{M}$ is constructed. If $\sigma_{1,i}$ is greater than $\sigma_{2,i}$, this means $s_{1,i}$ is focused in $I_{1}$ and the same region $s_{2,i}$ is de-focused in $I_{2}$. In the focus map $\bar{M}$, the focus value of pixels corresponding to $s_{1,i}$ is set to one, and it is set to zero if $\sigma_{1,i}≯\sigma_{2,i}$. That is,

(5) $$\underset{\forall \left(x,y\right)\in s_{i}}{\bar{M}(x,y)}=\left\{\begin{array}{cc} 1 & \mathrm{if}\;\sigma_{1,i}>\sigma_{2,i}\\ 0 & \mathrm{if}\;\sigma_{1,i}\leq \sigma_{2,i} \end{array}\right.$$

**Figure 4:**
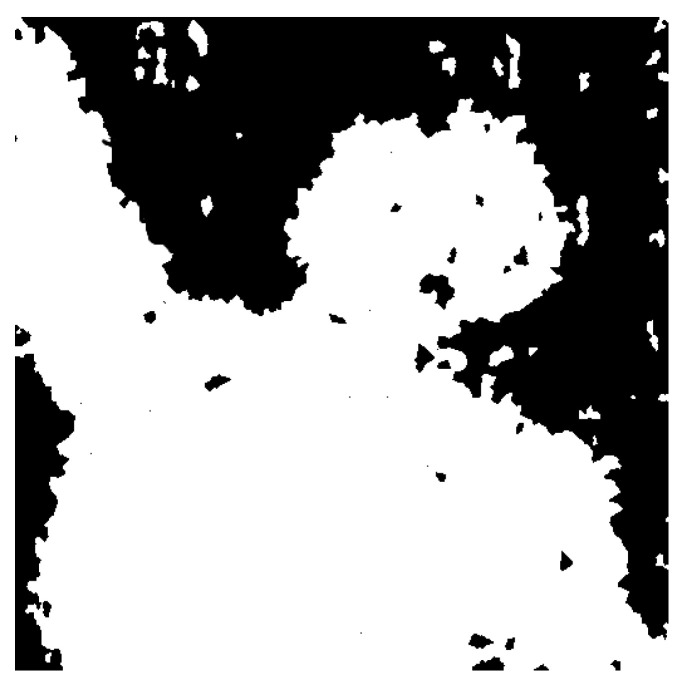
Initial focus map generated using the proposed strategy for the images shown in Figure 3.

Figure 4 shows the focus map constructed from the superpixels shown in Figure 3 using Equation (5).

It is possible that in the initial focus map obtained through the proposed algorithm, a few superpixels are incorrectly categorized, as can be noted in Figure 4. Some focused superpixels are falsely marked as de-focused, and a few de-focused patches are incorrectly marked as focused. These false positives and false negatives should be resolved to obtain a high-quality fused image. To this end, a neighbor constraint technique is proposed. We observed from experiments that the incorrectly categorized superpixels usually appear as a single entity, and most of its neighbors are correctly identified. Based on this observation, we form a neighbor constraint rule to eliminate the false positives and negatives from the map. The rule examines each superpixel in the map, investigates all its neighboring superpixels, and assigns the category that most of its neighbors have. Specifically, it is marked as focused if more than half of its neighbors are marked as focused, and vice versa.

**Figure 5:**
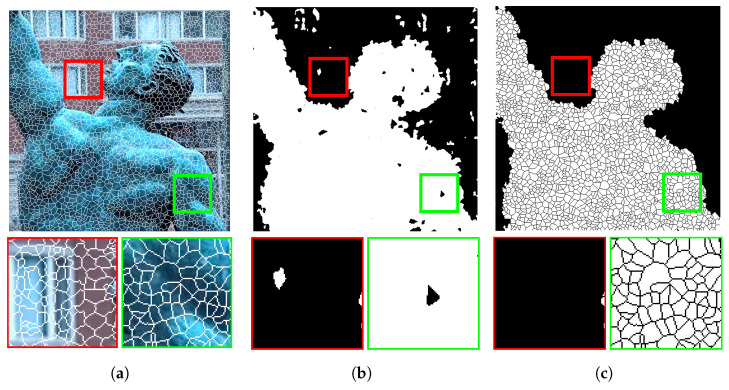
Initial focus map refinement: (**a**) a sample multi-focus image with superpixels structures overlaid on it, (**b**) the obtained focus map, and (**c**) the refined focus map.

Figure 5 shows the results of the proposed refinement process. Figure 5a shows a source image with superpixels overlaid on it; two sample focused and de-focused regions magnified to ease the inspection are shown below it. Figure 5b shows the initial focus map; superpixels structures are also overlaid to highlight the details, and selected regions are also shown below this figure. The results of the refinement procedure are shown in Figure 5c. The results show that most incorrectly marked regions were successfully recovered by the proposed refinement strategy.

### 3.3. Fusion Rule

**Figure 6:**
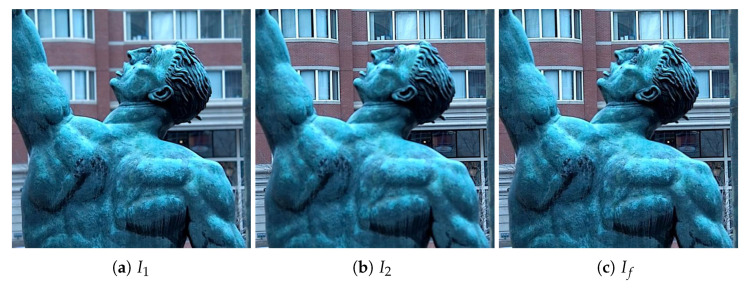
Fusion results using the refined focus map: (**a**) first source image $I_{1}$, (**b**) second source image $I_{2}$, and (**c**) fused image $I_{f}$.

The final focus map *M* obtained after refinement is used to fuse the contents of the source images $I_{1}$ and $I_{2}$ to generate the all-in-focus image $I_{f}$. The fusion rule simply takes the pixel value from $I_{1}$ if the focus map value is one and from $I_{2}$ otherwise. Figure 6 shows the fused image generated from the source images shown in Figure 3.

(6) $$I_{f}\left(x,y\right)=\left\{\begin{array}{cc} I_{1}\left(x,y\right) & \mathrm{if}\;M(x,y)=1\\ I_{2}\left(x,y\right) & \mathrm{if}\;M(x,y)=0 \end{array}\right.$$

## 4. Experiments and Results

**Figure 7:**
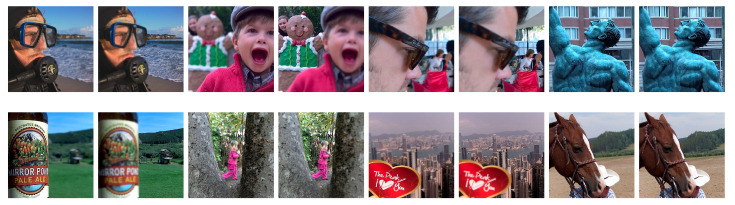
Sample multi-focus image pairs from the Lytro dataset.

In this section, we evaluate the performance of the proposed multi-focus image fusion method and compare it with the existing MIF techniques. The performance was assessed both qualitatively and quantitatively, and various analyses were also performed to evaluate the robustness of the proposed method. The experiments were performed on the recent widely used Lytro multi-focus dataset [43,70]. The dataset contains 20 color multi-focused image pairs, each of size 520×520. Sample image pairs from the dataset are shown in Figure 7.

### 4.1. Compared Methods

To evaluate the effectiveness of the proposed method, its performance was compared with existing well-known multi-focus image fusion (MIF) algorithms. In particular, nine representative methods were selected for the comparison. The discrete cosine harmonic wavelet transform (DCHWT) based method [24] reduces the fusion complexity by using the discrete cosine transform (DCT) and discrete harmonic wavelet coefficients. The wavelet based statistical sharpness measure (WSSM) [71] utilizes the spreading of the wavelet coefficient distribution as a sharpness measure and evaluates it by the adaptive Laplacian mixture model. The pulse coupled neural network (PCNN) method [26,72] uses the orientation information to obtain the fused image. The source images are decomposed into fixed-size blocks, and the image Laplacian of each block is computed to obtain feature maps that are used in PCNN for fusion. The DCTLP method [31] uses DCT coefficients and Laplacian pyramids to achieve fusion. The method presented in [73] uses Independent Component Analysis (ICA) and topographic independent component analysis bases for fusion.

In image fusion using luminance gradients (IFGD) [74], the highest gradients of each pixels’ locations of the source images are blended, and then, fused luminance is obtained by the reconstruction technique. In the nonsubsampled contourlet transform (NSCT) based MIF algorithm [27], NSCT decomposition is used to obtain the selection rules for sub-band coefficients. In the PCA method [75], the fused image is obtained using wavelets and principal component analysis (PCA). The CSR fusion algorithm [76] decomposes each source image into base and detail layers with the help of convolution sparse representation. It performs different fusion techniques on both layers and then merges the results. A deep convolutional neural network (CNN) based MIF method was presented in [54]. In particular, it uses the Siamese network for feature extraction and activity level measurement in source images.

### 4.2. Qualitative Performance Evaluation

The qualitative performance analysis was performed by analyzing the quality of fused images visually. For the visual inspection, we present the fusion results obtained by the compared methods and our algorithm. For comparison, “Swimmer” and “Cookie” image pairs are selected from the test dataset. The visual inspection is difficult to perform and generalize as each multi-focus image pair has different information about focused and de-focused regions. Nevertheless, it can certainly provide some insight into the quality of fused images obtained from different algorithms.

**Figure 8:**
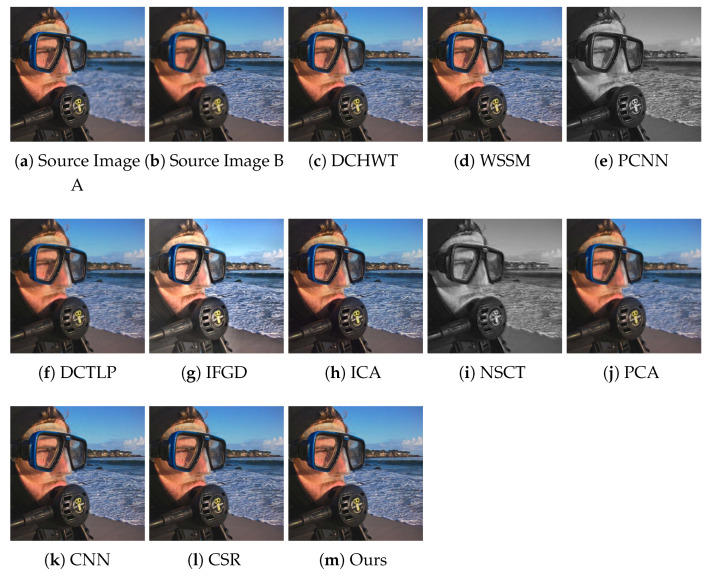
Visual quality assessment of the fusion results achieved by the proposed and the compared methods on the “Swimmer” multi-focus image pair from the test dataset. WSSM, wavelet based statistical sharpness measure; PCNN, pulse coupled neural network; DCTLP, discrete cosine transform Laplacian pyramid; IFGD, image fusion using luminance gradients; CSR, convolutional sparse representation.

**Figure 9:**
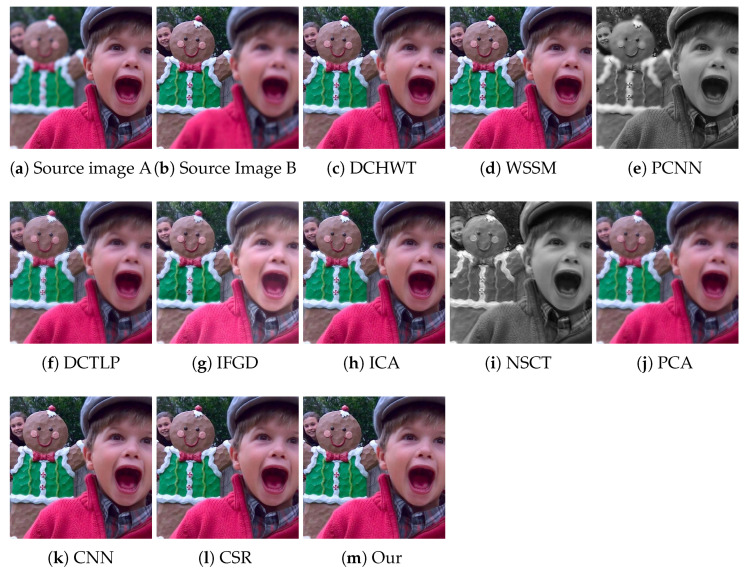
Visual quality assessment of the fusion results achieved by the proposed and the compared methods on the “Cookie” multi-focus image pair from the test dataset.

Figure 8 presents the fused images of the “Swimmer” image pair obtained by our algorithm and the compared methods. One can note that the results of the DCTLP method suffer from undesirable ringing artifacts: the fused image has lost most of the sharpness around the strong edges. Some blurred regions can be spotted in the results of the PCNN and NSCT methods. The fused image obtained by IFGD has lost some fine details due to extra brightness. The ghosting and ringing artifacts can be seen in the results of the PCA and NSCT algorithms as well. The ghosting artifact is also visible around the edges in the fused image produced by the ICA method. The fused images produced by WSSM, CNN, CSR, and our method are of good quality and are hard to visually differentiate. Similar observations can be made from the visual inspection of the results of ours and the compared methods on the “Cookie” image pair shown in Figure 9.

The visual quality inspection shows that the results produced by our method are crisp and are free of ghosting and ringing artifacts. A qualitative analysis, however, is difficult to perform due to various reasons including viewing conditions, trained subjects, etc., and it is particularly laborious on large datasets. Therefore, to truly assess the performance of an algorithm, along with the visual inspection, an objective fusion quality assessment is necessary.

### 4.3. Quantitative Performance Evaluation

The objective quality assessment of fused images is a tough task as the ground truth images of multi-focus images cannot be acquired. To objectively evaluate the performance of MIF algorithms, many metrics have been introduced, but none of them are certainly better than the others. Therefore, to evaluate the performance of fusion methods, it is desirable to use multiple metrics. These metrics evaluate the quality of the fused image with reference to the source multi-focus images. To perform an extensive objective performance evaluation of the proposed method and other competing techniques, we chose 12 fusion quality assessment metrics and computed the results over the whole dataset. These metrics assess the quality of a fused image using its different characteristics and modalities. Based on these characteristics, the metrics are generally grouped into four categories [8,77]: information theory based metrics, image structural similarity based metrics, image feature based metrics, and human perception based metrics. These metrics are summarized in Table 1.

- Information theory based metrics analyze the quality of the fused images by checking the amount of information transferred from the source images to the fused image. These metrics include: normalized mutual information (NMI) [79], visual information fidelity (VIFF) [81], and the nonlinear correlation information entropy metric $\left(Q_{NCIE}\right)$ [80].
- Image feature based metrics calculate the quality of fused image by analyzing the transferred features from the source images to the fused image. Gradient based fusion performance metrics $Q^{AB/F}$[82], $\left(Q_{G}\right)$[83], image fusion metric based on multiscale scheme $\left(Q_{M}\right)$[85], image fusion metric based on spatial frequency $\left(Q_{SF}\right)$[84], and image fusion metric based on phase congruency $\left(Q_{p}\right)$ [86,91] are examples of these metrics.
- Image structural similarity based metrics: The measurement of image similarity depends on the proof that the human visual system is profoundly adjusted to structural information, which is exploited in these metrics to assess the quality of a fused image. The loss of structural information is very important to estimate the image distortion. These metrics include Piella’s metric [87], Cvejie’s image fusion metric $\left(Q_{C}\right)$[88], and Yang’s image fusion metric $\left(Q_{Y}\right)$ [89].
- Human perception inspired fusion metrics, e.g., Chen Blum metric $Q_{CB}$ [90], measure the quality of fused images on the basis of the saliency map, which is generated by filtering and calculating the preservation of contrast.

The objective performance analysis of the proposed and the compared methods was carried out using all twelve metrics described in Table 1. Each metric’s scores was computed over the whole dataset using the implementation presented in [77], and the results were averaged to obtain an overall quality score. The comparison is presented in Table 2, and the best performing algorithm on each metric is marked in bold. The results show that for the widely used $Q^{AB/F}$ metric, our method performed the best, achieving the highest score 0.7539. The proposed method outperformed all competing techniques in most metrics including $Q^{AB/F}$, $Q_{G}$, $Q_{M}$, $Q_{SF}$, $Q_{Y}$, and $Q_{C}$. The performance of our method in the rest of the metrics was also appreciative, where it was the second or the third best algorithm. These statistics reveal that the proposed algorithm overall produces better quality fused images than most compared methods. Both qualitative and quantitative analyses reflect the effectiveness of the proposed method.

### 4.4. Computational Complexity

We also analyzed the computational complexity of the proposed and the compared methods. All MIF algorithms were executed over the whole dataset, and their average execution time was computed and is reported in Table 3. These times also include the file I/O time, if any, and were computed on an Intel^®^ Core™ i7 2.5 GHz processor with 8 GB RAM and 64 bit operating system. The results show that the PCA algorithm is the fastest among all approaches; the proposed method takes an average 122.61 seconds to generate a fused image. The main contributor to this time is the complex computation involved in estimating the superpixels and using the CIEDE2000 color distance model. An efficient implementation can help reduce its computational complexity.

### 4.5. Summary

The performance of the proposed MIF method was evaluated using different tools and techniques. To appreciate the performance of the proposed method, visual results achieved by the proposed and the compared MIF algorithms were presented in the preceding sections. From the visual inspection of the results presented in Figure 8 and Figure 9, we see that the results of most compared methods suffer from different impairments such as the ghosting effect, ringing artifacts, and blurry regions. In contrast, the results of the presented methods are crisp and free from these artifacts. The objective performance evaluation metrics confirm this. The advantages of the proposed method lie in its simplicity and ability to compute an accurate focus map. Unlike other competing methods that employ complex mechanisms to estimate the focus map and fusion strategies, the proposed method uses a simple fusion rule. We recall that the proposed MIF algorithm is built on the fact that the pixels in a small locality in an image are highly autocorrelated—having similar colors and textures. Based on this phenomenon, the pixels in the image are merged into larger patches, which provide the grounds for an accurate focus map, resulting in good fusion results. One limitation of the present proposed method is its inability to work with grayscale images. Since pixel color is an important clue in our focus map estimation method, its absence can degenerate its performance. However, extending it to grayscale images would certainly be an interesting investigation that we plan to carry out in the future.

## 5. Performance Analysis Using Different Color Distance Models and the Impact of other Parameters

In this section, we report different experiments and analyses that we performed to investigate the performance of the proposed image fusion algorithm. We performed three sets of experiments, first to evaluate the different color distance models for efficient superpixel estimation for fusion. The second set of experiments analyzed the impact of the number of superpixels on the performance of the proposed algorithm. In the third set of experiments, the contribution of color distance and spatial distance towards the overall distance measure was analyzed, and their impact on the performance of the proposed method was investigated.

### 5.1. Fusion Performance with Different Color Distance Models

We recall that determining the clusters to which the pixels are assigned to (Section 3.1) is decided using a distance measure $D_{s}$ Equation (3). $D_{s}$ is formed from the spatial distance $d_{s}$ and the color distance $d_{c}$ of the pixel and the cluster centers. The former measure Equation (1) is simple the Euclidean distance between the two coordinates, and the latter is the Euclidean distance between the lab colors of the two points Equation (2). We tested different color distance models to find the distance measure most suited for image fusion applications. In addition to the CIEDE2000 model (Section 3.1), the following color distance models are explored.

1. Euclidean color distance in the RGB color space: The simplest and the most well-known way to compute the distance between two colors is using the Euclidean distance in the RGB color space. The distance between two pixel colors $C_{1}\left(R_{1},G_{1},B_{1}\right)$ and $C_{2}\left(R_{2},G_{2},B_{2}\right)$ is computed as,
  (7) $$d=\sqrt{{\left(R_{2}-R_{1}\right)}^{2}+{\left(G_{2}-G_{1}\right)}^{2}+{\left(B_{2}-B_{1}\right)}^{2}}$$
2. Color approximation distance (CAD): The perception of brightness by the human eye is non-linear, and for each color, non-linearity is not the same, as proven by different experiments [92,93,94]. Therefore, there have been many attempts to weight the RGB values to better fit human perception so that the color approximation would be more appropriate. The CAD between two colors $C_{1}\left(R_{1},G_{1},B_{1}\right)$ and C2(R2,G2,B2) is computed as:
  (8) $$d=\sqrt{\left(2+\frac{\bar{r}}{256}\right)\Delta R^{2}+4\Delta G^{2}+\left(2+\frac{255-\bar{r}}{256}\right)\Delta B^{2}}$$
  where $\bar{r}$ is the average of $R_{1}$ and $R_{2}$; ΔR, ΔG, and ΔB are the differences between the red, green, and blue channels of the two colors, respectively.
3. CIEXYZ: The visualization of the RGB color space was not perfect because the color space of the human eye is greater than the experiment results of CIERGB, and an updated color difference model, CIEXYZ, was introduced by Commission Internationale de L’éclairage (CIE) [95]. It was mathematically designed to avoid the negative numbers known as tristimulus values. It consists of three parameters: non-negative cone response curve $\left(X\right)$, luminance $\left(Y\right)$, and partially-equal to blue color $\left(Z\right)$.
  $$\left[\begin{array}{c} X\\ Y\\ Z \end{array}\right]=\left[\begin{array}{ccc} 0.431 & 0.342 & 0.178\\ 0.222 & 0.707 & 0.071\\ 0.020 & 0.130 & 0.939 \end{array}\right]\times \left[\begin{array}{c} R\\ G\\ B \end{array}\right]$$
  The normalized tristimulus values x, y, and z were calculated from X, Y, and Z as: $\left[x\;y\;z\right]=\left[\frac{X}{S}\;\frac{Y}{S}\;\frac{Z}{S}\right],$ where S=X+Y+Z. The difference between the tristimulus values of two colors was then computed using the Euclidean distance formula.
4. CIE76: In the CIE76 color distance model, the CIELAB color space was used as it is considered to be the most exact means of representing color [96]. The color difference between two colors $C_{1}\left(L_{1},a_{1},b_{1}\right)$ and $C_{2}\left(L_{2},a_{2},b_{2}\right)$ in the CIELAB color space is calculated by following formula:
  (9) $$d=\sqrt{{\left(L_{1}-L_{2}\right)}^{2}+{\left(a_{1}-a_{2}\right)}^{2}+{\left(b_{1}-b_{2}\right)}^{2}}$$
  where $\Delta L^{*}=L_{1}-L_{2}$, $\Delta a^{*}=a_{1}-a_{2}$, and $\Delta b^{*}=b_{1}-b_{2}$
5. CIE94: The CIE94 color model retains the CIELAB color space, and it addressed the non-uniformities in CIE76 [97,98]. CIE94 was defined in the lightness, chroma, and hue (LCh) color space, and they are calculated from the Lab-coordinates.
6. CMC l:c: The color difference CMC (Colour Measurement Committee of the Society of Dyes and Colourists of Great Britain) l:c is calculated in the LCh color space and has two parameters: lightness (l) and chroma (c). For the application, it allows the users based on the ratio of l:c to weight the difference that is deemed appropriate.
7. CIEDE2000: The CIE Delta E (CIEDE2000) color distance model was introduced by CIE and provides an improved method for the calculation of color differences. A detailed discussion is presented in Section 3.1.

The proposed algorithm with each of these distance measures was executed on the whole test dataset. The quality of the fused images was evaluated using all 12 fusion quality assessment metrics, and the average results are presented in Table 4. The best scores for each metric are highlighted in bold. The results show that most fusion quality assessment metrics rate the fusion results of the proposed method with the CIEDE2000 color distance model as the best. In these experiments, all other parameters were kept fixed. Moreover, we also computed the average execution time of the proposed method with each color distance model, and the results are reported in Table 4. One can note that CIEDE2000 takes the maximum time of 122.61 seconds per image pair due to its complex computations. However, it produces better decision maps for fusion and therefore is preferred over other measures.

### 5.2. Impact of the Number of Superpixels on the Fusion Quality

**Figure 10:**
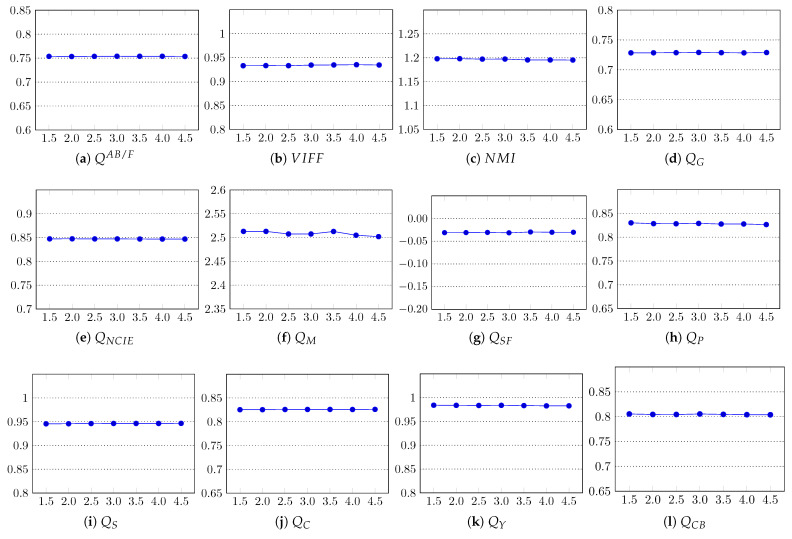
Impact of the number of superpixels *k* on the fusion quality of the proposed method. In all graphs, the x-axis represents *k* and the y-axis the quality score. The x-axis labels are coded as $10^{3}$.

The parameter that is important in the computation of superpixels is *k* (Section 3.1), the desired number of approximately equally-sized superpixels. For an image with *N* pixels, the size of each superpixel is approximately N/k pixels. In this experiment, we analyzed the impact of the number of superpixels on the performance of the proposed algorithm. We computed the fusion results with the proposed method using different values of *k*, and the results were evaluated using all 12 objective fusion quality metrics to get a general indicator. In particular, we tested the performance with k∈{1000,1500,2000,2500, 3000,3500,4000}. From the results shown in Figure 10, one can note that most metrics show negligible or no change in the quality of the fused images; therefore, the impact of *k* on the fusion performance of our method is negligible. However, using a larger value of *k* certainly increases the execution time of the method due to the extra computation needed in computing the superpixels and their subsequent steps. The average execution times for different *k* are reported in Table 5. In our method, k=3000 was set as the default.

### 5.3. Analysis of the Relative Contribution of Spatial and Color Distances

**Figure 11:**
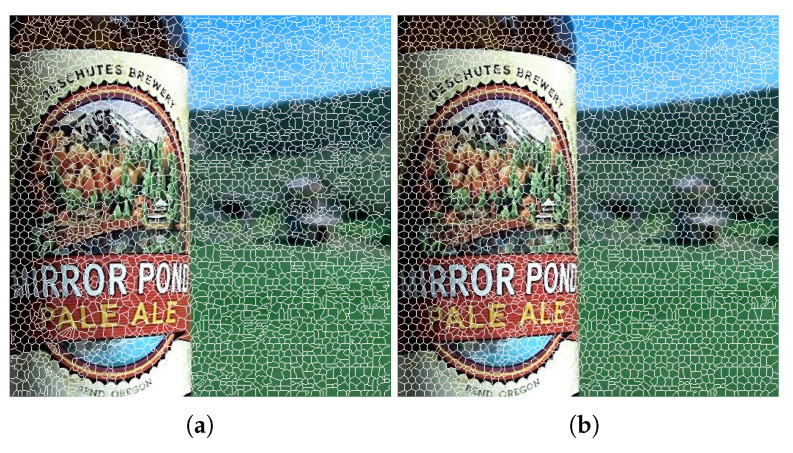
Superpixels estimated with the proposed algorithm using (**a**) m=15 and (**b**) m=40.

The parameter *m* in Equation (3) is used to control the compactness of the superpixels: it weights the relative information between the spatial and the color proximity. The recommended range of the value of *m* in the CIELAB color space is $\left[1,40\right]$. We performed a set of experiments using different values of weighting factor *m*. We note that for small values of *m*, the shape and size of the superpixels become less regular and more tightly adhere to the image boundaries. For a large value of *m*, the superpixels are more compact, and spatial proximity becomes more important. The results of superpixel estimations with different values of *m* on a sample image are shown in Figure 11.

**Figure 12:**
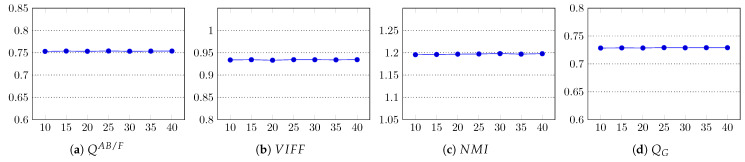

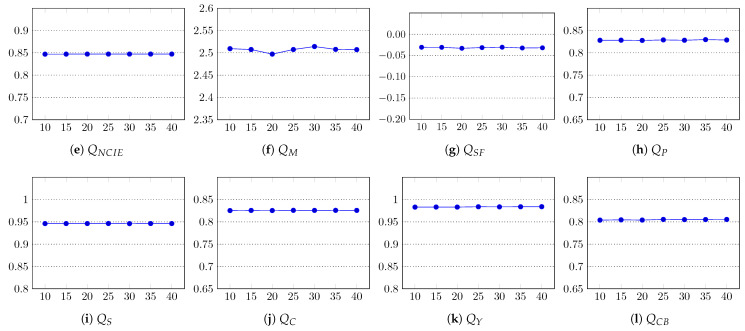
Impact of the weight factor *m* on the fusion quality of the proposed method. In all graphs, the x-axis represents *m* and the y-axis the quality score.

The fusion results were assessed using all 12 quality metrics, and their average scores are graphed in Figure 12. The results clearly show that *m* does not contribute to the quality enhancement of the fused image. Since for large values of *m*, the superpixels are more compact, the execution time of the method is reduced, as shown in Table 6. In the implementation of our method, the default value of *m* was set to 25.

## 6. Conclusions

In this paper, a novel multi-focus image fusion technique is presented. The proposed technique merges similar pixels into larger groups termed superpixels. The spatial and color properties of the pixels are exploited to estimate the superpixels in the source multi-focus images. Moreover, different color distance models are tested to obtain high quality fused images. Different statistical properties of the computed superpixels are analyzed to categorize them as focused and de-focused, and based on this information, a focus map is generated. Moreover, the wrongly categorized superpixels are corrected by a spatial constraint rule. The qualitative and quantitative experiments worked out on a benchmark multi-focus image dataset reveal that our method produces better quality images than existing similar techniques. The source code of the proposed method is released publicly at the project website (http://www.di.unito.it/~farid/Research/SBF.html, accessed on 18 February 2021).

## Figures and Tables

**Table 1:** Objective image fusion quality assessment metrics used in the performance evaluation.

Metric	Metric Description and Reference
Information theory based metrics	
NMI	Normalized mutual information [78,79]
$Q_{NCIE}$	Non-linear correlation metric [80]
VIFF	Visual information fidelity metric [81]
Feature based metrics	
$Q^{AB/F}$	Gradient based metric [82]
$Q_{G}$	Gradient based metric [83]
$Q_{SF}$	Spatial frequency based metric [84]
$Q_{M}$	Multi-scale scheme metric [85]
$Q_{P}$	Phase congruency based metric [86]
Structural similarity based metrics	
$Q_{S}$	Piella’s metric [87]
$Q_{C}$	Cvejie’s metric [88]
$Q_{Y}$	Yang’s metric [89]
Human perception based metrics	
$Q_{CB}$	Chen Blum’s metric [90]

**Table 2:** Objective performance evaluation of the proposed and the compared methods on the Lytro dataset. The best scores for each metric are highlighted in bold.

Metrics	DCHWT	WSSM	PCNN	DCTLP	IFGD	ICA	NSCT	PCA	CNN	CSR	Proposed
$Q^{AB/F}$	0.7212	0.7305	0.7036	0.6526	0.7174	0.7445	0.2314	0.5992	0.7514	0.7366	**0.7539**
VIFF	0.9021	0.9333	0.8565	0.8388	**1.0456**	0.9386	0.5851	0.7809	0.9548	0.9329	0.9344
NMI	0.9176	0.9748	**1.2068**	0.8296	0.5223	0.9374	0.5645	0.8939	1.0725	0.9921	1.1972
$Q_{G}$	0.6153	0.6758	0.6745	0.5390	0.6181	0.6787	0.2070	0.5340	0.7029	0.6447	**0.7291**
$Q_{NCIE}$	0.8291	0.8321	**0.8497**	0.8252	0.8145	0.8301	0.8159	0.8277	0.8384	0.8341	0.8472
$Q_{M}$	0.8821	0.9331	2.2555	0.6983	0.5633	1.0264	0.1796	0.4820	2.3431	1.3400	**2.5074**
$Q_{SF}$	−0.0900	−0.0899	−0.1050	−0.0941	−0.0744	−0.0684	−0.1522	−0.3890	−0.0321	−0.0490	**−0.0314**
$Q_{P}$	0.7839	0.8201	0.7483	0.6892	0.7632	0.8197	0.0486	0.7500	**0.8428**	0.8283	0.8289
$Q_{S}$	0.9477	0.9438	0.9133	0.9298	0.8901	**0.9547**	0.5802	0.9217	0.9485	0.9467	0.9463
$Q_{C}$	0.8019	0.8097	0.8183	0.7698	0.7485	**0.8334**	0.3853	0.7978	0.8253	0.7980	0.8258
$Q_{Y}$	0.9219	0.9567	0.9672	0.8747	0.8526	0.9515	0.3792	0.8490	0.9653	0.9340	**0.9838**
$Q_{CB}$	0.6977	0.7887	0.7476	0.6185	0.6118	0.7130	0.4778	0.6325	0.7816	0.7638	**0.8054**

**Table 3:** Execution time (seconds) comparison of the proposed and the compared MIF algorithms.

Method	DCHWT	WSSM	PCNN	DCTLP	IFGD	ICA	NSCT	PCA	CNN	CSR	Proposed
Time	9.39	215.49	1.31	0.33	1.01	11.30	174.00	0.04	106.32	345.76	122.61

**Table 4:** Comparison of different color distance models for the proposed multi-focus image fusion algorithm. The best values are in bold. Time (in seconds) is the average execution time of the proposed method with each color distance model. CAD, color approximation distance.

Metric	Euclidean	CAD	CIE76	CIE94	CIEXYZ	CMC	CIEDE2000
$Q^{AB/F}$	0.7483	0.7477	0.7520	0.7510	0.7491	0.7533	**0.7539**
VIFF	0.9192	0.9182	0.9256	0.9240	0.9182	0.9287	**0.9344**
NMI	1.1919	1.1919	1.1945	1.1953	1.1953	1.1946	**1.1972**
$Q_{G}$	0.7252	0.7242	0.7291	0.7287	0.7287	**0.7295**	0.7291
$Q_{NCIE}$	0.8478	**0.8479**	0.8476	0.8478	**0.8479**	0.8472	0.8472
$Q_{M}$	2.4243	2.4406	2.4445	2.4363	2.3985	2.4603	**2.5074**
$Q_{SF}$	−0.0403	−0.0387	−0.0393	−0.0412	−0.0457	−0.0335	**−0.0314**
$Q_{P}$	0.8085	0.8077	0.8239	0.8239	0.8214	0.8224	**0.8289**
$Q_{S}$	0.9449	0.9449	0.9465	0.9463	0.9456	**0.9467**	0.9463
$Q_{C}$	0.8199	0.8190	0.8240	0.8238	0.8226	0.8250	**0.8258**
$Q_{Y}$	0.9803	0.9796	0.9827	0.9825	0.9822	0.9826	**0.9838**
$Q_{CB}$	0.7904	0.7881	0.8006	0.7994	0.7950	0.8005	**0.8054**
Time	52.08	56.87	44.71	45.31	**40.94**	53.67	122.61

**Table 5:** Execution time of the proposed method with different numbers of superpixels *k*. Time (in seconds) is the average execution time. The best time is marked in bold.

*k*	1500	2000	2500	3000	3500	4000	4500
Time	**97.86**	109.16	118.69	122.61	119.98	132.39	131.43

**Table 6:** Impact of weight factor *m* on execution time. Time (in seconds) is the average execution time of the proposed method with different values of *m*. The best values are in bold.

*m*	10	15	20	25	30	35	40
Time	151.72	141.16	125.76	122.61	110.32	110.32	**98.54**

## Data Availability

Data sharing not applicable.
